# Gamma‐aminobutyric acid production by *Lactobacillus brevis* A3: Optimization of production, antioxidant potential, cell toxicity, and antimicrobial activity

**DOI:** 10.1002/fsn3.1838

**Published:** 2020-08-20

**Authors:** Behrooz Alizadeh Behbahani, Hossein Jooyandeh, Fereshteh Falah, Alireza Vasiee

**Affiliations:** ^1^ Department of Food Science and Technology Faculty of Animal Science and Food Technology Agricultural Sciences and Natural Resources University of Khuzestan Mollasani Iran; ^2^ Department of Food Science and Technology Faculty of Agriculture Ferdowsi University of Mashhad Mashhad Iran

**Keywords:** gamma‐aminobutyric acid, high‐performance liquid chromatography, *Lactobacillus brevis*, whey powder

## Abstract

In this study, whey powder was used as the basic compound for fermentation culture and the production of bioactive gamma‐aminobutyric acid (GABA) compound. GABA is a nonprotein four‐carbon amino acid that inhibits stress signals by preventing brain signals, reducing stress, and being effective in treating neurological disorders and decreasing the growth of cancer cells. Due to the side effects caused by the chemical type of GABA, the biological production of GABA has attracted. Three levels of whey powder (5%, 10%, and 15%), and monosodium glutamate (MSG) (1%, 3%, and 5%) were selected at temperatures (25, 30, and 37°C) and after fermentation, the presence of GABA in the culture medium was examined by thin‐layer chromatography. The optimal amount of GABA was measured by using high‐performance liquid chromatography. The results of the central composite design of the response surface methodology at a significant level of 95% showed that the optimal treatment was 14.96% whey powder, 4.95% MSG at temperature of 37°C and fermentation for 48 hr and under these conditions, GABA production was 553.5 ppm. The results of the fermented extract tests showed that the highest antimicrobial activity was on *Escherichia coli* and the highest free radical scavenging was 59.67%. The IC_50_ level in the Caco‐2 cancer cell cytotoxicity test was 39.5 mg/ml. According to the results, the combination of whey with MSG can be used as a cheap substrate to produce a valuable bioactive GABA product, and the cellular extract of this fermentation can also be used as an antimicrobial and antioxidant compound in food and pharmaceutical formulations.

## Graphical abstract

According to the results, the combination of whey with monosodium glutamate can be used as a cheap substrate to produce a valuable bioactive GABA product, and the cellular extract of this fermentation can also be used as an antimicrobial and antioxidant compound in food and pharmaceutical formulations.

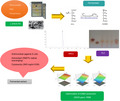

## 1 INTRODUCTION

Gamma‐aminobutyric acid, commonly known as GABA, is a nonprotein amino acid and is the major neurotransmitter inhibitor in the brain. GABA improves brain cell metabolism by increasing oxygen delivery and blood flow, and is involved in regulating growth hormone secretion, protein production, fat burning, and lowering blood pressure. GABA is a drug with antioxidant, antidepressant, anti‐insomnia, and pain‐relieving effects and is used to treat autoimmune diseases, stroke, neurological disorders, and diabetes control. GABA with high bio‐activity potential can be used in the production of functional foods. Many microorganisms are able to produce GABA in their cells due to the activity of the enzyme glutamic acid decarboxylase (GAD). Some of them have the ability to produce high levels of GABA in the presence of glutamate, pyridoxyl‐5‐phosphate, and acidic conditions (Dhakal, Bajpai, & Baek, 2012).

As the population grew and more energy resources were needed, various technologies thought to solve the problem of lack of resources. One of these solutions is the use of waste from different factories in the production of useful and usable products. Many of these sources contain various compounds, including carbohydrates, proteins, vitamins, and so on. Therefore, the use of food industry waste to produce valuable products such as amino acids seems to be a good way to reduce production costs and waste of food resources. Dairy plants as a strong wastewater with high oxygen concentration are a biological and chemical requirement that contains a lot of organic matter. This type of waste causes serious problems in the municipal wastewater treatment system. In addition to environmental problems caused by dairy industries, the presence of solids in milk along with effluent indicates the loss of a valuable product from the dairy industry. By recycling and reusing these types of effluents, these disadvantages would be partially compensated (Tikariha & Sahu, 2014).

Dairy industry is one of the largest producers of effluent, one of the most famous of which is the liquid released from the clots created during cheese production, which contains high amounts of organic compounds such as carbohydrates and proteins. It can be used as a source of carbon and nitrogen by microorganisms. Whey is a by‐product of hard, semihard, soft, and casein‐produced cheeses produced by the Rennet enzyme. Whey has a pH 4.5 and since it has a high biochemical oxygen demand, it is not possible to apply it on agricultural fields. On the other hand, it is a very nutritious source of various proteins, carbohydrates, and many types of salts. So technology and recycling are essential. Therefore, this compound can be used as a cheap and useful culture medium for the growth of microorganisms and the production of various products such as amino acids, exopolysaccharides, enzymes, organic acids, and so on (Baldasso, Barros, & Tessaro, 2011; Tikariha & Sahu, 2014).

*Lactobacillus* is a type of lactic acid bacteria that is considered as a safe bacterium. These bacteria are gram‐positive, nonsporadic, catalase‐negative, and usually immobile. They are mostly seen in the form of rods, but other shapes such as coccobacilli, carina, and strings can also be recorded. The optimum temperature for the growth of this genus is 30–40°C and the percentage of organic bases of cytosine and guanine is low. This genus is commonly found in traditional fermented foods and is also found in the gastrointestinal tract and makes up most of the small intestinal flora. Important species of this genus include *L. plantarum*,* L. fermentum*, *L. pentosus*,* L. brevis*, and* L. casei*. According to various studies, some *L. brevis* strains have the ability to produce GABA in nitrogen and carbon sources (Ramos, Thorsen, Schwan, & Jespersen, 2013).

Glutamic acid or its salts, pH, temperature, and compounds of the culture medium, including carbon and nitrogen sources, are the main factors in the production of GABA by microorganisms. Other culturing agents can be optimized based on the biochemical properties of the enzyme produced by GABA (by GAD). Adding glutamate can increase GABA production in cultivated environments (Su, Schlicht, & Gänzle, 2011).

The aim of this study was to use different percentages of aquifer base culture medium as a carbon source and glutamic acid treatments at three different fermentation temperatures to optimize GABA production. The antimicrobial, antioxidant, and toxicity properties of fermented cellular extract cells were also investigated.

## 2 MATERIALS AND METHODS

### 2.1 Materials

This experimental performed from July 2019 to March 2020. The chemicals used in this study include culture medium de man, rogosa and sharpe (MRS), mueller hinton agar (MHA) and mueller hinton broth (MHB), compounds such as iron chloride, butylated hydroxy anisole (BHA), triphenyltetrazolium chloride, sodium acetate, tetrahydrofuran potassium hydroxide, trichloroacetic acid, and ammonium sulfate were purchased from Merck, Germany. Dulbecco’s modified eagle medium (DMEM), fetal bovine serum (FBS), and penicillin and streptomycin antibiotics were developed from Gibco.

### 2.2 Activation of *L. brevis* A3

In the present study, the native *L. brevis* A3, isolated from Horreh fermented food, (isolated and identified by molecular methods in Vasiee, Alizadeh Behbahani, Tabatabaei Yazdi, Mortazavi, and Noorbakhsh (2018)), was used to investigate its ability to produce GABA. In order to activate, the lyophilized bacterium was transferred to the MRS Broth culture medium and heated at 24°C for 24 hr. Activated microorganisms were transferred to the MRS agar culture medium and heated at 24°C for 24 hr. This culture medium was used as a storage medium for subsequent tests. The purity of the desired strain was investigated and confirmed by hot staining (Vasiee et al., 2018).

### 2.3 Fermentation

#### 2.3.1 Preparation of medium culture

The whey used in this study was prepared from Pegah Dairy Factory, Iran. The production of whey powder was in order to achieve the highest production efficiency, solubility, and the lowest amount of moisture and mass density with 39.37% dry matter and in the dryer with an initial temperature of 250°C. To evaluate the production of GABA, three levels of whey powder and three levels of MSG powder along with three levels of fermentation temperature were selected and prepared according to the statistical design of treatments in volume of 50 ml. Magnet and hot plate were used to improve the solubility of the samples. All samples were passed through a vacuum filter with filter paper 541 and the filtered solution was autoclaved and sterilized for 15 min at 12°C (Franciosi et al., 2015).

#### 2.3.2 Fermentation process

According to the statistical design, 100 μl of activated *L. brevis* A3 was transferred to Falcon containing 5 ml of the prepared culture medium at pH of 6 and placed in the incubator for 48 hr (Kook & Cho, 2013).

### 2.4 Evaluation of GABA production

#### 2.4.1 Thin‐layer chromatography/Spectrophotometry

After 48 hr of fermentation, the supernatant was centrifuged at a temperature of 4°C with 11180 *g* for 10 min. To eliminate possible microorganisms, the surface phase was filtered by a 0.22 µ filter. To evaluate the production of GABA, an active silica gel plate with dimensions of 20 × 20 cm was used. According to Kook & Cho, 2013 method, the plate was horizontally at a distance of 2 cm from the bottom, with a pencil in one direction at points by distance of 1 cm was marked. Then, the plate was blotted with a 2‐microliter capillary tube. In this test, pure GABA solutions and bacterial culture medium were used separately as control. A solution consisting of ninhydrin with a combination of butanol, acetic acid, and distilled water in a ratio of 2:3:5 was used as the mobile phase. After the band was identified, the screen was placed in the 70°C oven for 80 min. Then bands in front of the GABA were cut and dissolved in 75% ethanol solution and 0.6% copper sulfate (2:38) separately. The mixture was then placed in a 2795 *g* incubator for 40 min at a temperature of 40°C. The absorption of each sample was read at 512 nm (ethanol and copper sulfate solution were used to reset the spectrophotometer). According to the adsorbents read by the solution containing pure GABA and the corresponding graph and equation, only a small amount of GABA was obtained in the fermented extract of the samples (Karimi, Yazdi, Mortazavi, Shahabi‐Ghahfarrokhi, & Chamani, 2020; Kook & Cho, 2013).

#### 2.4.2 High‐performance liquid chromatography

After finding the optimal treatment with the TLC method, the GABA level in the optimal treatment was measured by the high‐performance liquid chromatography (HPLC) device based on the method of Liu, Li, Zhou, Chen, and Tu (2015). The sample for 15 min at 1006 *g* was centrifuged and supernatant passed through a 0.45 µ filter. The filtered sample was added to 0.2 mM sodium bicarbonate at pH 9.8 in a 9:1 ratio. Then, 8 g/L dansyl chloride derivative was added to the sample and placed for 1 hr in 30°C (no light). GABA standards were prepared and derived at 250 and 450 mg/L concentrations. Analysis was performed using HPLC equipped with column C18 (250 × 4.6 mm). The mobile phase consists of two phases A (sodium acetate 50 mM, methanol, tetrahydrofuran at ratio (5:75:42) and phase B (methanol) which were mixed with linear gradient from 1% to 100%. The mobile phase with flow rate 1 ml/min was injected into the column, and a UV detector at a wavelength of 254 nm at temperature of 40°C was used to identify the output material of the column (Liu et al., 2015; Wu & Shah, 2015).

### 2.5 Investigation of fermented extract properties in optimum conditions

#### 2.5.1 Antimicrobial potential against food spoilage pathogenic bacteria

To prepare the bacterial extract for antimicrobial activity, the optimal treatment extract was first filtered to remove bacterial cells at 2795 *g* for min 20 at 5°C centrifuged and supernatant passed through 0.22 μm filter paper and using NaOH 1N the pH was reached to 7, and finally lubricated with a BETA LCS plus 2–8 freeze dryer under freezing at −45°C and heated to 32°C under 0.38 mbar vacuum for 40 hr. The dried sample was dissolved again with 4 ml of sterile distilled water, and its antimicrobial properties were investigated by minimum inhibitory concentration (MIC) and well diffusion agar. The pathogenic strains used in this test included *Escherichia coli* ATCC 25922, *Staphylococcus aureus* ATCC 25923, *Pseudomonas aeruginosa* PTCC 1707, *Salmonella typhimurium* PTCC1609, and *Listeria innocua* ATCC 33090 (Alizadeh Behbahani, Falah, Arab, Vasiee, & Yazdi, 2020; Falah et al., 2019).

##### Minimum inhibitory concentration

In this method, 100 μl of different concentrations of the optimum sample in MHB culture medium (350, 400, 450, 500, 550, 600, 600, and 650 mg/ml) along with 10 μl of microbial suspension was added to 96‐well microplates. At the end of the incubation, 10 μl of triphenyl tetrazolium chloride 5% were added to each of the wells and reincubated. After 30 min in the first well when the red color was not visible, the concentration of that well was recorded as the MIC of the samples (Matuschek, Åhman, Webster, & Kahlmeter, 2018).

##### Well diffusion agar method

Suspensions of pathogenic bacteria were diluted according to the McFarland Standard and then cultured in MHA plates. Wells in 6.8 mm diameter were then created into the plates by the end of the sterile pipette. After that, 110 μl of fermented extract was added to the wells. After 48 hr incubation at a temperature of 37°C, the diameter of the nongrowing circle around each cavity was measured. MHA plates (without fermentation extract) were used as positive control (Tabatabaei Yazdi, Behbahani, Vasiee, Ali, & Yazdi, 2015).

#### 2.5.2 Antioxidant properties

Antioxidant activity of optimum sample was studied by using two methods of reduction power and radical scavenging activity (DPPH) 2,2‐diphenyl‐1‐picrylhydrazyl.

##### Reducing power

In order to evaluate the regenerative strength of the optimum sample, 1 ml of the sample was mixed with 1 ml of distilled water and 1 ml of potassium ferrocyanide (1%), and the resulting solution was stirred. After heating the solution for 20 min at 50°C in a bain‐marie, 2.5 ml of trichloroacetic acid was added to and stirred again. The solution was centrifuged at 63 *g* for 5 min. Then, 2 ml of supernatant was mixed with 2 ml of distilled water and 1 ml of ferric chloride (FeCl_3_) 0.1% and after all mixing and storage for 10 min at room temperature, the adsorption of the solution was recorded at 700 nm. The results were reported as absorption unit (Alizadeh Behbahani, Noshad, & Falah, 2019a, 2019b; Nooshkam et al., 2019).

##### DPPH‐Radical Scavenging (DPPH‐RS) activity

In order to evaluate the optimal free radical inhibitory activity, 2 ml of the sample was mixed with 1 ml of 0.2 mmol DPPH radical solution in 95% ethanol. After keeping the sample for 30 min at room temperature and in a dark place, its absorption was recorded at 517 nm (Equation 1):

(1) $$\text{DPPH}\;\text{radical}\;\text{scavenging}\;\text{activity}\;\left(\%\right)\;=\;\left(1‐\frac{\text{Sample}\;\text{absorbance}\;}{\text{Control}\;\text{absorbance}}\right)\times 100$$

Ethanol BHT solution with a concentration of 15 mg/ml was used as the standard antioxidant and to compare the antioxidant activity of the samples (Oliveira et al., 2016).

#### 2.5.3 Cytotoxic properties

The toxicity of the optimal treatment of fermented extract against Caco‐2 cancer cell was examined by MTT method (MTT assay: 3‐ (4.5‐dimethylthiazol‐2‐yl) ‐2.5‐diphenyltetrazolium bromide). After cell culture in microplate 96‐wells, and when cells reached at least 70% cell growth, it was replaced with fresh DMEM and FBS culture medium and concentrations of zero to 600 mg/ml of extract. Fermented was added. MTT cell survival was investigated based on MTT approach. After 24 hr of incubation, the MTT solution was added to the wells and incubated in an incubator which contained CO_2_ for 3 hr. After removing the samples and adding DMSO, the absorption of the samples at 570 nm was investigated by ELISA method. The concentration that is able to kill 50% of cells is referred to as IC_50_ (Alizadeh Behbahani et al., 2020; Alizadeh Behbahani, Noshad, & Falah, 2019a, 2019b).

### 2.6 Optimization by response surface methodology

The statistical design in this study was the response level method using the central compound design central composite design (CCD) to optimize the variables affecting the dependent variable (GABA production). The mean GABA produced from three repetitions of each experiment was considered as dependent or response variables. The design of the experiments, the analysis of the data, and the drawing of the graphs were done by modeling the second degree of Design‐Expert® 8.0.0 software by Stat‐Ease, Inc.

## 3 RESULT AND DISCUSSION

### 3.1 Optimization of fermentation and GABA production

The beginning of fermentation due to glucose uptake and organic acid production pH reduced and after some time due to H+ consumption as a result of GABA production, the pH increases logarithmically. In acidic pH conditions between 4 and 5, due to increased GAD activity, GABA production increases so that pH reduction can be considered as a criterion for evaluating enzyme activity. GABA production begins in the growth phase of bacterial (log phase) and close to the stationary phase due to increased GAD enzyme activity, production increases. It is an intracellular enzyme that is produced in response to acidic conditions. In most lactic acid bacteria, its active form is dimer, but in *L. brevis* it is tetramer (Shelp et al., 2012).

According to the results, whey is a usable substrate for the growth of bacteria, which can also be used in fermentation cultures. That part of the waste plants with the highest dry matter content is called whey, which has a good potential to become a valuable product, regardless of the environmental pollution. The main compounds in whey are carbon, nitrogen, and phosphorus, so it can be used in fermentation cultures. Due to the physical condition of this compound as well as the combination of the culture medium compounds, the drying process was done by spray dryer. Drying is aimed at carefully controlling the properties of the powder and creating favorable conditions for estimating different needs as well as product uniformity. Spray drying is a suitable method for heat‐sensitive biological materials such as protein compounds (enzymes, etc.) with minimal structural and functional damage (Lavari, Páez, Cuatrin, Reinheimer, & Vinderola, 2014).

**Figure 1:**
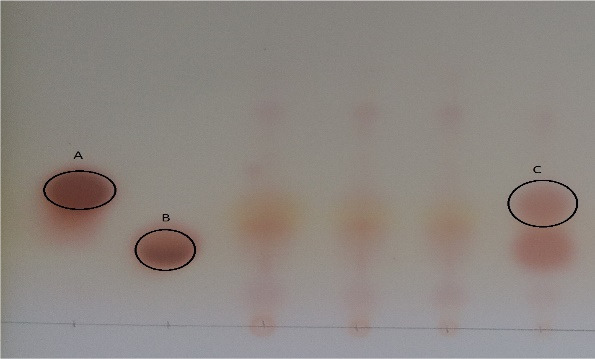
Thin‐layer chromatography: Standard concentrations—ppm GABA (a), MSG (b), optimum sample (c)

Thin‐layer chromatography (TLC) does not require expensive equipment and is suitable for simultaneous analysis of a large number of samples, so it is recommended to analyze the production rate of GABA (Figure 1). In this method, ninhydrin is used as the most important compound to identify GABA. Ninhydrin releases amines and carboxyl. Amino acid binds to and separates ninhydrin through a pair of free nitrogen amines. Then, by removing a carbon dioxide molecule and binding the carboxyl group to decarboxylation, the amino acid is converted to an aldehyde compound and the ninhydrin itself is converted to hydrindantin, forming a pink Roman complex (Basu & Sinhababu, 2014).

In order to determine the trend of changes in GABA production, and to examine the effect of each of the independent variables, it is first necessary to determine the appropriate model to fit the test data. Statistically, a suitable model is the one that its nonfit test is not significant and the adjusted *R* ^2^ and *R* ^2^ have the highest value. Due to the significance of the fitness test for production as well as the adjusted *R* ^2^ and *R* ^2^ values, the second‐order multi‐component model was evaluated and selected to evaluate the trend of change in responses. To determine the response level model, the result of linear, second‐order responses and the interaction of independent variables were used. In Equation 2, the experimental relationship between GABA production efficiency and independent variables with real numbers is shown:

(2) $$\text{GABA}\;=\;257.86\;+\;154A\;+\;55B\;+\;9C\;+\;40\text{AB}\;‐\;2.5\text{AC}\;+\;12.5\text{BC}\;+\;51.59A^{2}\;‐\;3.41B^{2}‐23.41C^{2}$$

In which A, B, and C are linear effects, A^2^, B2, and C^2^ are the effects of squares and AC, BC, and AB are interaction effects.

Because carbon has a direct effect on the yield, bacterial composition, structure, and properties, it is the most important compound in the culture medium used to produce microbial metabolites. Lactose is the main carbohydrate in whey, the main nutrient for growth and production, and is an alternative source of carbon for growth and production. It seems that the reason for the effect of the carbon source on production is the high demand of the strains to the carbon source. In a study by Shan et al. (2015), among the LAB, *L. brevis* was able to produce GABA in a culture medium containing date waste and whey milk. In this study, it was found that carbohydrate compounds are converted to alpha‐ketoglutarate and ammonia by being in the carb cycle and then produced in the cytoplasm of L‐glutamate cells. L‐glutamate is converted to di‐glutamate and eventually to polyglottic acid. GABA is produced during the decarboxylation of glutamic acid (Shan et al., 2015).

**Figure 2:**
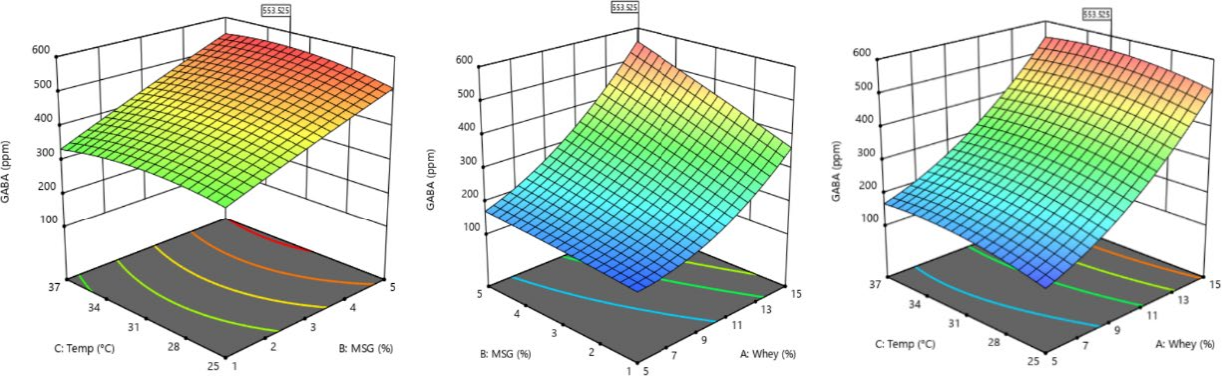
Three‐dimensional curve of treatments interaction effects on GABA production

Glutamic acid is the substrate for the decarboxylation reaction of GABA production, and it is obvious that with increase of its percentage, the amount of GABA production will increase. According to a study by Zhang et al. (2012), products containing MSG, due to their short‐chain and bioactive peptides and proteins available to microorganisms, can be used in fermentation culture medium to produce various amino acids. The yield of GABA production in Dhakal et al Research (2012) was from the combination of fermented soybean extract and whole milk and whey milk in the MRS medium and inoculation of about 500 ppm *L. brevis*. In this method, GABA production was performed by HPLC method and bacterial growth was performed using spectrophotometry and cell absorption measurement. Zhuang et al., 2018 examined the effect of culture medium compounds on the growth of *L. brevis* bacteria. The highest production (about 40 ppm) in the culture medium was carbon source (molasses), MSG 5%, and magnesium sulfate, and the rate of production in 60 hr of fermentation and initial pH was 5.5 at 32°C. As shown in Figure 2, with increasing percentages of whey, GABA production increased with a gentle slope; as a result, with the increase in the percentage of whey to about 15%, the trend of increasing GABA production was higher, which was due to the effect of carbon source on increasing fermentation efficiency (Dhakal et al., 2012; Zhang et al., 2012; Zhuang et al., 2018).

As shown in Figure 2, with increase in MSG concentration, GABA production increased linearly. In general, under low concentration of whey and MSG, the production efficiency is significantly lower. Maximum GABA production was observed at the maximum MSG concentration. The results show that the GABA production improves with increasing carbon and nitrogen sources. Given that these two energy sources are the main component of GABA production, further production seems to be related to the increase in carbon and nitrogen sources to the consumption of these resources in the next part of cultivation (stationary phase). In this study, the production of GABA was considered at higher MSG concentrations, and it may be inferred that the ideal concentration for GABA production depends on factors such as substrate, growth conditions, and type of microorganism. GABA synthesis in lactic acid bacteria is catalyzed by the enzyme glutamate decarboxylase, and GABA production depends on the biochemical properties of the enzyme (Moo‐Chang et al., 2010). The production of the GAD in response to acidic conditions is induced in LAB and reacts to glutamate as a substrate, during which it converts it to GABA and carbon dioxide. Glutamate, as an enzyme substrate, is a major parameter affecting GABA production during fermentation. Therefore, adding glutamate to the fermentation environment increases the production of GABA in GABA‐producing bacteria. As shown in Figure 2, with increasing the concentration of MSG in the fermentation medium, the slope of GABA production increased. These results confirm the increased activity of the GAD in the presence of MSG.

Wu and Shah (2015) examined the effect of concentrations of 1%, 3%, 5%, and 7% of MSG on GABA production by lactobacillus strains isolated from kimchi and observed that although the rate of conversion of MSG to GABA with increase in concentration decreased, but the final production continued to increase with increasing concentration. These results were observed in the study of Zhang et al. (2012). An increase in GABA production was also observed due to an increase in the concentration of sodium glutamate in *L. paracasei* and *L. Bruce* NCL912. Suwanmanon and Hsieh (2014) used whey to grow *L. plantarum* and studied the production of amino acids in this culture medium. The maximum production of amino acids was observed in the logarithmic phase of cell growth. The bacterium then enters the stationary phase and the production rate decreases. Limitations of carbon, nitrogen, phosphate, and oxygen are factors that affect the conversion of a carbon source to an amino acid. The lower the carbon to nitrogen ratio, the higher the substrate degradation (Suwanmanon & Hsieh, 2014; Wu & Shah, 2015; Zhang et al., 2012).

Glutamic acid decarboxylase is produced from the same subunits with molecular mass in the range of 54–62 kDa, whose catalytic amino acid strands contain a highly protected lysine root. Although the carboxylation reaction for GAD is similar in LAB, the initial structure in the N‐terminal and C‐terminal regions of the enzyme varies considerably in different directions. Differences in the initial structure of the enzyme can affect the ability of the enzyme to different concentrations of substrate in GABA production (Zhuang et al., 2018).

Incubation temperature is one of the most important factors influencing the maximum production of GABA in the fermentation process. In addition to biocatalytic activity, temperature also affects the thermodynamic equilibrium of reactions. On the other hand, the high‐yield conversion of glutamate to GABA is directly related to cell density, which also depends on the appropriate cultivation temperature (Dhakal et al., 2012).

As shown in Figure 2, with temperature change to 37°C, the production of GABA with a gentle slope increased. Previous research has shown that GABA production depends on temperature, but the optimum temperature range was usually between 30 and 40°C. The temperature dependence of GABA production is not only affected by the species of bacteria but also by the composition of the culture medium. The optimum temperature for GABA production by *L. plantarum* DSM19463 is in the range of 30–35°C, in culture medium containing soybean and calcium carbonate, *L. bochneri*, 30°C (Shan et al.,^2015^), and in a culture medium containing yeast extract and skim milk, *L. paracase* NFRI 7415, 37°C (Suwanmanon & Hsieh, 2014) has been reported (Shan et al., 2015; Suwanmanon & Hsieh, 2014).

**Figure 3:**
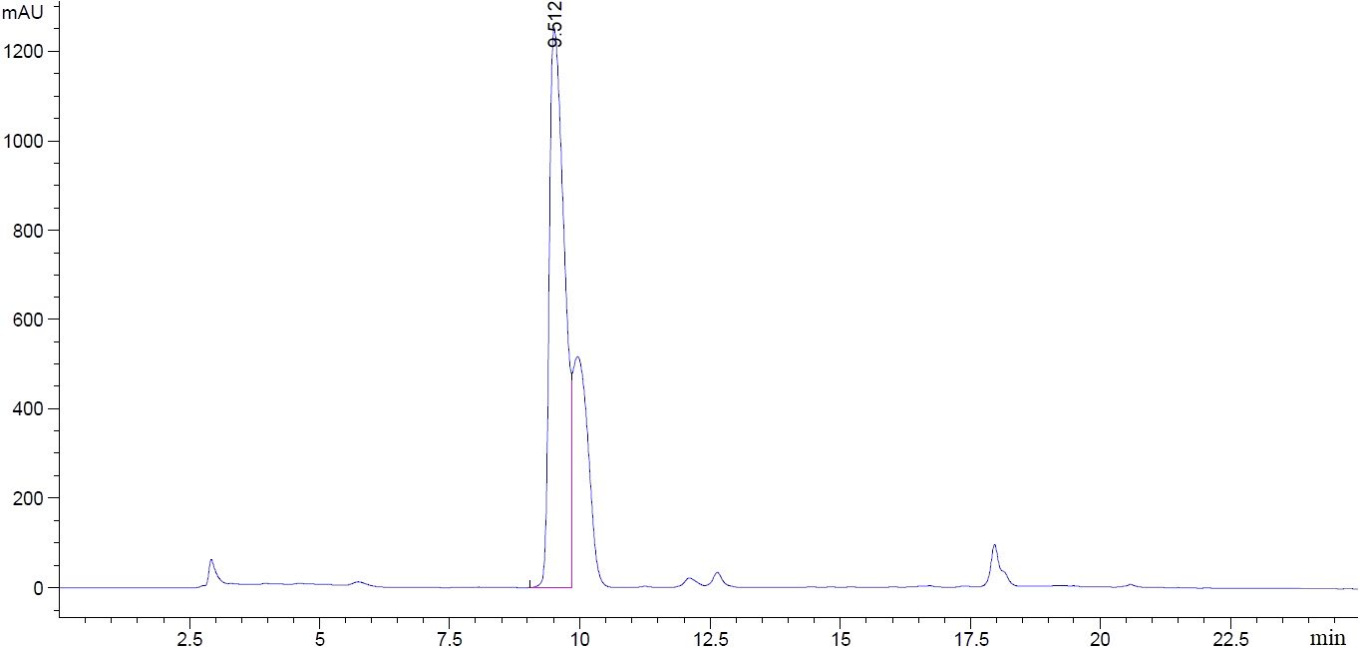
Optimal treatment HPLC

In order to optimize the fermentation conditions based on the production of GABA, the upper and lower limits and the optimum of each of the characteristics and weight and their importance were determined. The amount of GABA produced by *L. brevis* in the medium containing 14.96% whey with 4.95% MSG at 37°C and 48.5 mg/L fermentation for 48 hr was 553 mg/L. To validate the model, optimal point validation experiments were performed in laboratory conditions by remeasuring the efficiency of amino acid production and comparing it with the efficiency of the model using HPLC method. The maximum production output was about 500 ppm. Therefore, considering the prediction of the efficiency of the model in optimal points with numerical value, the high degree of adaptation of the experimental efficiency with the predicted efficiency can be observed. According to GABA's internal standard injection, the average inhibition time for GABA was 9 min and 51 s. In chromatograms (Figure 3), there are a number of uncertain peaks that are related to other compounds used in the derivative stage of the sample. After specification of the standard curve and GABA’s concentration, consequently the linear regression equation( y = 435.03x ‐ 431.077) with coefficient of R2 > 0.9997 was determined.

Process optimization is one of the most important functions in today’s competitive industry. In order to mass‐produce the factors affecting the production of amino acids, it is also necessary to optimize the industry for its commercial production. On the other hand, the high cost of research requires the use of methods that make it possible to determine the variables that affect a process with the least number of experiments, which is done using classical methods and designing statistical models (Shan et al., 2015). In this study, it was found that the response surface methodology can be used to find the optimal conditions of the system, taking into account the variables affecting the response. Additional tests were performed to make better use of the optimal treatment.

### 3.2 Properties of fermented extract in optimum conditions

#### 3.2.1 Antimicrobial properties

**Figure 4:**
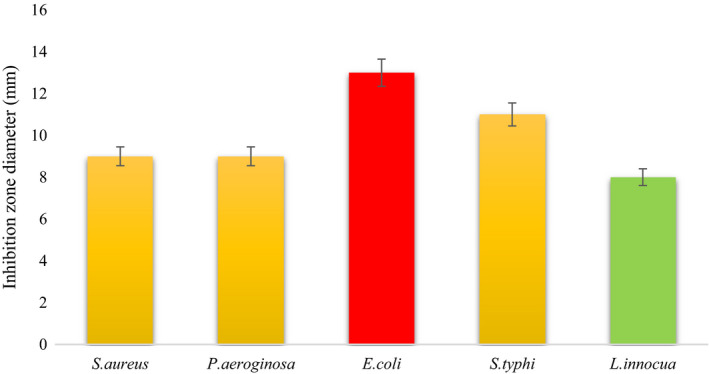
Evaluation of the antimicrobial properties by measuring inhibition zone diameter against some pathogenic bacteria by well diffusion agar method

The MIC of fermented extract was depending on the type of microorganisms. *S. typhi* was the most susceptible bacteria with a MIC of 450 mg/ml, and *L. innocua* was the most resistant bacteria. In the well diffusion method, *S. typhi* and *E. coli* had a significant inhibition zone diameter (11 and 13 mm) (Figure 4). Benítez‐Serrano et al. (2018) confirmed the antimicrobial activity of fermented extract of probiotic strains *L. brevis* and *L. plantarum* in skim milk compared with *E. coli* and *S. typhi*. The researchers linked the antimicrobial activity of fermented extract to the production of bacteriocins and their stability response to acidic conditions. Park et al. (2012) reported that the antimicrobial activity of fermented wheat extract by probiotic *L. plantarum* on *Streptococcus mutans* was due to the effect of lipopeptides produced during fermentation (Benítez‐Serrano et al., 2018; Park et al., 2012). In a study by Adak, Upadrasta, Kumar, Soni, and Banerjee (2011), protein hydrolysis by probiotic bacteria led to the formation of high‐hydrolysis proteins and low molecular weight peptides that could act as antimicrobial peptides. According to a study by Ramos et al. (2013), cationic antimicrobial peptides have the potential to bind to lipopolysaccharides with negative charge (in gram‐negative bacteria) and teichoic and lipoteichoic acids (in gram‐positive bacteria), so part of the antimicrobial activity is observed. Can be attributed to it (Adak et al., 2011; Ramos et al., 2013; Tabatabaei Yazdi et al., 2015).

#### 3.2.2 Antioxidant properties

Investigating reducing power as an effective antioxidant method to assess the ability of an electron to donate electrons is an antioxidant compound. This method is based on the reduction of ferro‐ferrocyanide chloride‐iron complex in the form of ferrous iron by antioxidants. The optimum sample had significant regenerative power, but showed less antioxidant activity compared with BHA (Table 1). The results of the present study were consistent with the findings of Zareie, Tabatabaei Yazdi, and Mortazavi (2019). The reducing power of the optimum sample can be due to the large number of hydrogen ions produced during fermentation. In a study by Nooshkam et al. (2019), it was shown that decarboxylation and enzymatic hydrolysis lead to the breaking, opening, and subsequent emergence of amino acids with the ability to donate electrons. These amino acids can react with free radicals to form relatively more stable compounds and stop free radical chain reactions. Low molecular weight peptides have been reported to have a high ability to neutralize DPPH radicals. In addition, the presence of tyrosine amino acids in the C‐terminal peptide has been shown to be essential for the radical neutralizing effect of some peptides (Alizadeh Behbahani, Noshad, & Falah, 2019a, 2019b; Nooshkam et al., 2019; Zareie et al., 2019).

**Table 1:** Antioxidant properties of fermented extract

Antioxidant properties	Optimization	BHA
Reducing power	0.57 ± 0.01	1.28 ± 0.83
DPPH	59.67 ± 0.79	75.53 ± 2.16

In various studies, the antioxidant effect of polysaccharide conjugates has been reported. Examining the antioxidant effect of Millard reaction products, dairy‐glucose compounds stated that the melanoidins produced during the reaction increased the browning and regenerative power of conjugates, and the hydroxyl and pyrrole groups of Millard products could act as reducing agents. Oliveira et al. (2016) in a study on the conjugates antioxidant properties from whey protein and some sugars, identified hydroxyl groups as a reducing agent and stated that these products play an important role in breaking down radical chains by donating electrons (Oliveira et al., 2016).

#### 3.2.3 Cytotoxicity

**Figure 5:**
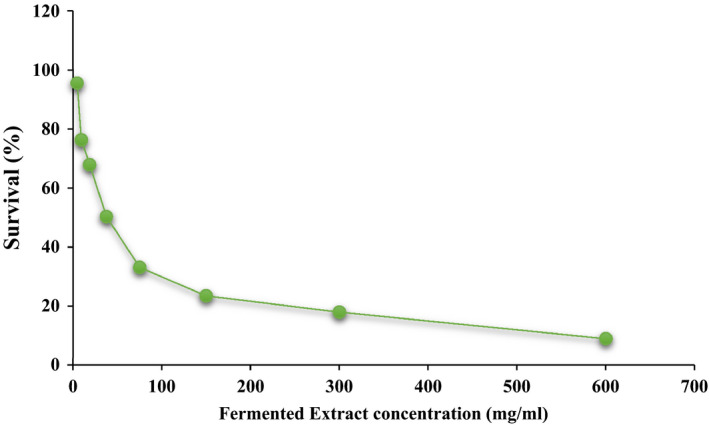
Cell viability at different concentrations of fermented extract

MTT approach was used to investigate cell toxicity. This method is based on the breakdown of tetrazolium salt by the enzyme succinate dehydrogenase, the mitochondria of living cells. Figure 5 shows the cellular toxicity of fermented extract after 24 hr on Caco‐2 cancer cell. Cellular toxicity depended on the concentration and was higher at higher concentrations. The IC_50_ sample was 39.5 mg/ml. According to Adak et al. (2011) study on dichloromethane extract on epithelial cells, the cause of cell toxicity was a change in the polarity of cancer cells and mitochondrial membranes and the destruction of cell ATP and protein crossings. IC_50_ was reported to be 18 mg/ml. The toxicity of the cancer cell is attributed to the production of probiotic fermentation extracts to the production of carbohydrates and organic acids such as lactic and butyric, as well as the production of bioactive compounds such as GABA during fermentation, which can lead to antimutagenic properties (Adak et al., 2011; Alizadeh Behbahani, Noshad, & Falah, 2019a, 2019b). The presence of probiotic strains and the strengthening of the immune system and the inactivation of effective enzymes in mutagenicity, the production of short‐chain fatty acids during fermentation, as well as the activation of the protective enzyme glutathione transferase 2 all lead to inactivation of the toxic cell. Adak et al. (2011) found that balancing beta‐glucuronidase enzymes and decreasing pH during fermentation reduced clone cancer cells.

## 4 CONCLUSION

Advances in understanding the relation between nutrition and health to achieve optimal health and reduce the risk of disease have led to the development of high‐fat foods. In this study, the maximum production of GABA using *L. brevis* A3 strain was obtained in culture medium containing 14.96% whey with 4.95% MSG at 37°C and for 48 hr at 53.5 ppm. The production of many amino acids by various chemical, enzymatic, etc. methods is costly, so if you use cheap raw materials in a biological way, the cost of production can be greatly reduced. The findings show that the biological production of GABA by lactic acid bacteria, in addition to its benefits, is biologically active, safe and environmentally friendly, and that it provides the production of new products to enrich with. Due to the biological nature of GABA produced in this study, it is possible to provide a substitute for the chemical type in pharmaceuticals by purifying this amino acid. Also, by performing clinical tests on fermented extract containing GABA produced, it will be possible to produce functional food products. In addition, the rational use of industrial waste can help to reduce the environmental impact of the dairy industry.

## CONFLICT OF INTEREST

The authors have declared no conflict of interest.

## ETHICAL APPROVAL

This article does not contain any studies with human or animal subjects.
